# Vitamin K deficiency: a case report and review of current guidelines

**DOI:** 10.1186/s13052-018-0474-0

**Published:** 2018-03-14

**Authors:** Maria Rosaria Marchili, Elisa Santoro, Alessandra Marchesi, Simona Bianchi, Lelia Rotondi Aufiero, Alberto Villani

**Affiliations:** 1 Pediatric and Infectious Disease Unit, Bambino Gesu’ Children’s Hospital, IRCCS, Piazza Sant’Onofrio 4, Rome, Italy; 2 Pediatric Department, University of Tor Vergata, Rome, Italy

**Keywords:** Vitamin K, Deficiency, Bleeding, Breastfeeding, Case report

## Abstract

**Background:**

Vitamin K, a fat soluble vitamin, is a necessary cofactor for the activation of coagulation factors II, VII, IX, X, and protein C and S. In neonatal period, vitamin K deficiency may lead to Vitamin K Deficiency Bleeding (VKDB).

**Case presentation:**

We present the case of a 2 months and 20 days Caucasian male, presented for bleeding from the injections sites of vaccines. At birth oral vitamin K prophylaxis was administered. Neonatal period was normal. He was exclusively breastfed and received a daily oral supplementation with 25 μg of vitamin K. A late onset vitamin K deficiency bleeding was suspected. Intravenous Vitamin K was administered with complete recovery.

**Conclusions:**

Nevertheless the oral prophylaxis, our case developed a VKDB: it is necessary to revise the current guidelines in order to standardize timing and dosage in different clinical conditions.

## Background

Vitamin K, a fat soluble vitamin, is a necessary cofactor for the activation of coagulation factors II, VII, IX, X, and protein C and S. Vitamin K deficiency may lead to Vitamin K Deficiency Bleeding (VKDB). The VKDB is a disorder of hemostasis in which coagulation parameters are quickly corrected by vitamin K supplementation. The term VKDB replaced the wording of hemorrhagic disease of the newborn since vitamin K deficiency bleeding may also occur in the postnatal period.

The diagnosis is suggested by an International Normalized Ratio (INR) ≥ 4 or a Prothrombin Time (PT) greater than 4 times the normal values, in the presence of a normal platelet count and normal fibrinogen level. Diagnosis confirmation requires vitamin K-dependent factors dosage, which levels are quickly corrected by parenteral administration of 1 mg of vitamin K [1].

There are different forms of VKDB: early, classic and late.

Early form occurs within the first 24 h of life in infants born from mothers treated during pregnancy with anticonvulsants (carbamazepine, phenytoin and barbiturates), antituberculosis drugs (isoniazid, rifampicin), some antibiotics (cephalosporins) or vitamin K antagonists (warfarin) and who did not received vitamin K prophylaxis before the delivery [1]. It incidence in at-risk neonates without vitamin K supplementation varies from 6% to 12%. The classic form occurs between 24 h to 7 days of life and is more often idiopathic; in term born not receiving vitamin K prophylaxis it presents an incidence of 0,25–1,5% in older reviews [2] and 0,01–0,44% in more recent reviews [3]. The classic form is related to the low placental transfer of vitamin K, low concentration in breast milk, lack of gastrointestinal flora in the newborn gut, and poor oral intake that commonly occurs in the newborn period as breastfeeding is initiated. The late form occurs between the 2nd week and the 6th month of life, with a peak between 3 and 8 weeks after birth; it has an incidence of 1/15.000–1/20.000 births and it is typical of exclusive breastfed infants or newborns with malabsorption or cholestasis because vitamin K absorption is closely dependent on the intestinal availability of bile. The hemorrhagic manifestations mainly involve gastrointestinal tract and skin, but also the central nervous system in late forms [4, 5]. Late VKDB carries a significant morbidity and mortality rate, with a mortality as high as 20–50% and a morbidity characterized by neurologic defects including hydrocephalus, cerebral atrophy, encephalopathy, epilepsy, and developmental delay [6].

Breastfeeding has been implicated as risk factor for the development of VKDB because human milk vitamin K concentration (median 2.5 mg/L [0.85–9.2 mg/L]) is significantly lower than currently available formula milk (4–25 mg/100 kcal approximately corresponding to 24–175 mg/L) [7, 8]. On average daily vitamin K intake of breastfed infants is < 1 mg within the first 6 months of life, whereas the intake of formula-fed infants is on average up to 100 times higher [9].

Vitamin K deficiency leads to the synthesis of under-carboxylated proteins called PIVKA (protein induced by vitamin K absence), that are unable to bind calcium and therefore inactive. PIVKAs are released from the liver into the blood and their level increases with the severity of the deficiency. PIVKAs are, however, much more commonly reported in breastfed infants [10].

## Case presentation

We reported the case of a 2 months and 20 days white Caucasian male, presented for bleeding from the injections sites of the first dose of hexavalent and pneumococcal vaccine.

He was born from unrelated parents at 41 weeks of gestational age by urgent cesarean section, with a birth weight of 3200 kg and a 5-min Apgar score of 10. His mother reported no use of drug during pregnancy and a previous miscarriage. At birth oral vitamin K prophylaxis was administered. Neonatal period was normal. He was exclusively breastfed and he received a daily supplementation of 25 μg of vitamin K and 400 IU of vitamin D.

At admission he was in fairly good general conditions, he was awake and responsive, with a valid crying and age-appropriate neurological findings. On the anterior region of both thighs, the injection sites of vaccines were recognizable, with a slight bleeding from the injection site on the left leg, without signs of edema. He had a normal cardiorespiratory activity and normal vital signs (blood pressure 70/46 mmHg, heart rate 126 bpm, respiratory rate 30 acts/min, body temperature 36 °C).

Blood tests showed a progressive anemia with a minimum value of hemoglobin of 7.8 g/dl (laboratory findings are shown in Table 1). The dosage of coagulation factors showed low values of the vitamin-K dependent factors (factor II 2%, factor VII 4%, factor IX 2%, factor X 5%) and normal values of factor V (128%). Abdominal and brain ultrasound excluded an ongoing bleeding. Fecal elastase on three samples and serology for HIV, HCV, HBV, CMV, EBV, HHV-6, Parvovirus B19 were negative (except for positive IgG for CMV).

**Table 1:** laboratory findings at admission

Test	Result	
	At admission	At discharge
Hb	7,8 g/dl	9,8 g/dl
PLT	152.000/uL	328.000/uL
INR	Not measurable	0,94
PT	> 100 s (n.v. 11,5-15,3)	12,4 (n.v. 11,5-15,3)
aPTTs	166,9 s (n.v. 26-50)	28,5 (n.v. 26-50)
aPPTr	5,72 (n.v. 0,88-1,69)	0,98 (n.v. 0,88-1,69)
Antithrombin III	126% (n.v. 32-72)	/
Fibrinogen	467 mg/dl (n.v. 200-500)	414 (n.v. 200-500)
Factor II	2% (n.v. 30-60)	/
Factor VII	4% (n.v. 40-72)	/
Factor IX	2% (n.v. 20-36)	/
Factor X	5% (n.v. 40-72)	/
Factor V	128% (n.v. 70-120)	/
AST	78 UI/L (n.v. 5-40)	69 UI/L (n.v. 5-40)
ALT	41 UI/L (n.v. 5-40)	40 UI/L (n.v. 5-40)

*N.v*. normal values, *Hb* hemoglobin, *PLT* platelets, *INR* International normalized ratio, *PT* Prothrombin Time, *aPTT* Activated partial thromboplastin time, *AST* Aspartate-aminotransferase, *ALT* alanine-aminotransferase

Based on the clinical history and laboratory findings, a vitamin K deficiency bleeding “late onset” was suspected. Intravenous Vitamin K (Konakion 10 mg) was administered together with a continuous infusion of 1 g of tranexamic acid. Considering the low hemoglobin values, he was transfused without any adverse reaction.

After vitamin K administration a normalization of the coagulation parameters with persistence of anemia (Hb 8.8 g/dl) was observed. Treatment was therefore continued with oral vitamin K, and iron and folic acid supplementation.

During hospitalization, he did not presented further bleeding or other symptom. At discharge coagulation parameters were normal (PT 12.4 s, INR 0.94, aPTTs 28.5 s, aPTT-r 0.98, PLT 328,000/μl, fibrinogen 414 mg/dl) and hemoglobin values were increased (Hb 9.8 g/dl). The child was discharged with the indication to continue oral vitamin K supplementation with Konakion 10 mg twice weekly until weaning.

## Discussion

Since 1961 the American Academy of Pediatrics has recommended that all neonates receive a single intramuscular dose of 0.5-1 mg of vitamin K for the prevention of VKDB [11].

NICE guidelines of 2015 reiterated the recommendation to administer vitamin K at birth; in particular, they recommended to administer 1 mg of vitamin K intramuscularly to all newborns in order to prevent VKDB. If intramuscular administration is rejected by parents, the administration of multiple oral doses is recommended. These recommendations are based on the evidence that intramuscular administration of vitamin K is clinically more effective than oral administration, and that multiple oral doses of Vitamin K are necessary to obtain adequate protection against the late VKDB onset in breastfed children [12, 13].

A Joint Position Statement from the Canadian Paediatric Society and the College of Family Physicians of Canada, published in 1997 and re-affirmed in 2014, recommended that a single intramuscular dose of vitamin K (0.5 mg for birthweight ≤1500 g or 1.0 mg for birthweight ≥1500 g) should be administered to all newborns within the first 6 h after birth. If intramuscular vitamin K is refused by parents, an oral dose of 2 mg vitamin K was recommended at the time of first feeding, followed by a second dose at 2 to 4 weeks, and a third dose at 6 to 8 weeks [14]. Similar recommendations are provided by two practice guidelines, one from Perinatal Services British Columbia [15] and one from the Winnipeg Regional Health Authority [16].

In 1992 Golding et al. reported an increased risk of developing childhood cancer after intramuscular vitamin K prophylaxis [17]. Subsequent studies, however, showed no significant correlation between parenteral vitamin K prophylaxis at birth and development of tumors in childhood [18, 19]. But since the report of Golding et al. an increasing trend towards oral vitamin K administration had become.

A single vitamin K oral dose at birth is efficacy in prevention of classic VKDB, but it does not protect against late VKDB because of the shorter duration of oral vitamin K activity compared to intramuscular administration. In fact studies suggest that after a single oral dose vitamin K levels fall to unsupplemented levels within 2 weeks. Several mechanism can explain the major efficacy of intramuscular vitamin K administration, including poor absorption after oral administration and depot effect at the site of intramuscular injection [20].

Although it is clear that more than a single oral dose is needed, especially in exclusively breastfed infants, there is no consensus in the literature regarding the best oral vitamin K regimen to prevent late VKDB. In the absence of evidence there is a heterogeneity in prophylactic strategies, concerning the route of administration and the dosage of vitamin K used, with differences not only between countries, but also within the same country, as in the case of Italy. So a variety of continuous oral regimens have been studied (Table 2), but clinical trials comparing their efficacy and safety are lacking.

**Table 2:** VKDB prophylaxis strategies in various countries

Country	Years Studied	Oral Vitamin K dosing regimen	Incidence of Late VKDB per 100,000 Infants (95% CI)
Netherlands	1992-1994	1 mg at birth; 25 μg daily for 13 wk.	0 (0 to 0.7)
	since 2005	1 mg at birth; 25 μg daily for 13 wk.	3.2 (1.2 to 6.9)
Germany	1993-1994	3 × 1 mg (days 1, 4-10, and 28-42)	1.3 (0.8 to 2.0)
	1995-1998	3 × 2 mg (days 1, 4-10, and 28-42)	0.4 (0.2 to 0.7)
	1997-2000	3 × 2 mg (days 1, 4-10, and 28-42)	0.8 (0.4 to 1.4)
	1997-2001	3 × 2 mg (days 1, 4-10, and 28-42)	0.44 (0.2 to 0.9)
France	/	2 mg weekly for 6 mo	no date available
Australia	1993-1994	3 × 1 mg (days 1, 3-5, and 21-28)	1.5 (0.5 to 3.6)
Denmark	1992-2000	2 mg at birth; 1 mg weekly for 3 mo	0 (0 to 0.9)
	since 2000	2 mg im at birth	no date available
Switzerland	1995-2002	2 × 2 mg (h 4, day 4)	1.2 (0 to 6.5)
	since 2003	3 × 2 mg (h 4, day 4, week 4)	0,87 (0.24 to 2.24)
United Kingdom	/	1 mg im at birth; 3 × 1 mg po (day 1, week 1, week 4)	0.1
	/	3 × 2 mg po (day 1, week 1, week 4)	0.43
Canada	/	1 mg im at birth	0.37
United States	/	1 mg im at birth	no date available
Italy	/	various schemes	no date available

Primarily epidemiological data from several European countries showed that the most effective dosing strategy seems to be a continuous oral prophylaxis with weekly doses of 1 mg (Danish scheme) or daily doses of 25 μg (Dutch scheme) for 3 months. In particular, Danish scheme is based on an important study of Hansen et al., published in 2003, which has established that 1 mg of oral vitamin K given weekly during the first 3 months of life, following an initial oral dose of 2 mg of vitamin K directly after birth, is as effective as 1 mg of vitamin K given intramuscularly at birth [21]. Afterwards a surveillance study of 2005, published in 2008, in Netherland evidenced that daily dose of 25 μg was not effective in preventing idiopathic late VKDB. For this reason the authors suggested to double the daily dose of vitamin K to 50 μg for all breastfed babies from 1 to 13 weeks to prevent bleeding in also infants with underlying cholestatic liver disease [22].

Recently Witt et al. evaluated whether a prophylactic regimen of 1 mg vitamin K orally at birth followed by 150 μg daily during weeks 2 to 13 sufficiently prevented VKDB in breastfed infants. Their data in high-risk group, for example undiagnosed children with biliary atresia, showed that this regimen does not successfully prevent VKDB in these children, in contrast to a regimen consisting of a single intramuscular injection of 2 mg vitamin K at birth. They concluded that a prophylactic regimen for breastfed infants consisting of 1 mg vitamin K orally at birth, followed by either 25 μg or 150 μg daily during weeks 2 to 13, does not sufficiently prevent VKDB in breastfed infants with still undiagnosed biliary atresia. They assumed that this insufficient prevention is also present in infants with yet undiagnosed others forms of neonatal cholestasis. In their study efficient prevention was obtained by regimen consisting of single intramuscular injection of 2 mg vitamin K at birth [23].

However, cases of VKDB in exclusively breastfed healthy newborns, despite intramuscular prophylaxis at birth, have been described, suggesting that prophylaxis with a single dose of intramuscular vitamin K may be inadequate to prevent all cases of late VKDB [24–26].

In consideration of these reports, since 2004, the Italian Society of Neonatology recommends an oral administration of vitamin K in all exclusively breastfed newborns in the period from the 2nd to 14th weeks of age [27].

In our clinical case the child had been subjected at birth to oral administration of vitamin K and, subsequently, to a daily oral supplementation with 25 μg. Nevertheless, he developed a late-onset VKDB, although he had not cholestatic disease or malabsorption, but exclusively breastfeeding as only risk factor.

The reporting of late-onset VKDB in breastfed children invite us to reflect on the problem of prophylaxis in breastfed infants and the need of reviewing the guidelines and, in particular, the dose of oral supplementation with vitamin K in children taking only breast milk, considering, in addition, that hat oral supplementation is dependent on the compliance of the parents administering the vitamin in form of drops (with the real risk that the assumed dose is less than the desired dose).

Finally, despite evidence of major efficacy of intramuscular administration in preventing late forms of VKDB, mostly in children with risk factors (breastfeeding, unknown conditions of cholestasis or malabsorption), in the absence of shared guidelines there are still some birth Centers where oral prophylaxis is routinely performed at birth.

## Conclusion

NICE guidelines of 2015 recommend to administer 1 mg of vitamin K intramuscularly to all newborns in order to prevent VKDB. If intramuscular administration is rejected by parents, the administration of multiple oral doses is recommended as a second option. These recommendations are based on the evidence that intramuscular administration is clinically more effective than oral administration, and that multiple oral doses of vitamin K are necessary to obtain adequate protection against late onset VKDB in breastfed children [12, 13].

Nevertheless the exact dosage and timing of oral administration after the starting dose at birth, are not yet well defined [28]. On the other hand, late-onset VKDB cases have also been reported in children undergoing intramuscular administration of vitamin K at birth (not followed by subsequent oral supplementation) [24–26].

No data are available regarding the incidence of late onset VKDB after intramuscular prophylaxis at birth followed by oral supplementation [29].

Studies should be performed to evaluate the incidence of late onset VKDB in children who receive prophylaxis with vitamin K intramuscularly at birth, compared to those who receive it orally, with the same successive regimen of oral supplementation.

On the basis of such results we think that it will be necessary a review of guidelines about VKDB prophylaxis in order to standardize timing and dosage in different clinical conditions.

## Abbreviations

CNS: Central nervous system
INR: International normal ratio
NICE: National Institute for Health and Care Excellence
PIVKA: Protein induced by vitamin K absence
PT: Prothrombin Time
VKDB: Vitamin K deficiency bleeding
